# Nerve injury and neuropathic pain — A question of age

**DOI:** 10.1016/j.expneurol.2015.07.013

**Published:** 2016-01

**Authors:** Maria Fitzgerald, Rebecca McKelvey

**Affiliations:** Department of Neuroscience, Physiology & Pharmacology, University College London, London WC1E 6BT, United Kingdom

**Keywords:** Neonatal, Paediatric, Peripheral nerve, Neuroimmune, Microglia, Neuropathic pain, Adolescent, Dorsal horn, Pro-inflammatory, Anti-inflammatory, Somatosensory, Plasticity

## Abstract

The effects of peripheral nerve injury on somatosensory processing and pain are highly dependent upon the age at which the damage occurs. Adult nerve injury rapidly triggers neuropathic pain, but this is not so if the same nerve injury is performed in animals below postnatal day (P) 28, consistent with observations in paediatric patients. However, longitudinal studies show that pain hypersensitivity emerges later in life, when the animal reaches adolescence, an observation that could be of clinical importance. Here we discuss the evidence that the central consequences of nerve damage are critically determined by the status of neuroimmune regulation at different ages. In the first postnatal weeks, when spinal somatosensory circuits are undergoing synaptic reorganisation, the ‘default’ neuroimmune response is skewed in an anti-inflammatory direction, suppressing the excitation of dorsal horn neurons and preventing the onset of neuropathic pain. As animals grow up and the central nervous system matures, the neuroimmune profile shifts in a pro-inflammatory direction, unmasking a ‘latent’ pain response to an earlier nerve injury. The data predicts that nerve injury in infancy and childhood could go unnoticed at the time, but emerge as clinically ‘unexplained’ or ‘functional’ pain in adolescence.

## 1 Introduction — The ontogeny of neuropathic pain

### 1.1 The first phase — No neuropathic pain

Peripheral nerve damage in early life does not simply remove a source of sensory input from the somatosensory system, it triggers great change in neural circuitry and leads to long term alterations spinal somatosensory function. However, the nature of these changes is dependent upon when exactly, in terms of postnatal age, this nerve damage occurs.

A major consequence of nerve damage, in adult man and laboratory animals, is the onset of neuropathic pain, characterised by allodynia and pain hypersensitivity from the partially denervated regions (Gilron et al., 2015). Thus, spared nerve injury (SNI), a classic partial denervation model (Decosterd and Woolf, 2000), produces robust mechanical allodynia, measured as a fall in hindpaw cutaneous sensory threshold to 16% of controls, within one postoperative day and lasting at least 28 days (Howard et al., 2005).

Strikingly, neuropathic pain does not arise if exactly the same nerve injury is performed in young animals. Rat pups aged 3, 10 and 21 days at the time of surgery do not display equivalent allodynia at any time up to 28 days later (Howard et al., 2005). Only at postnatal day (P) 33 does SNI lead to a significant and persistent allodynia with the threshold falling to 55% of control values. A comparable lack of neuropathic pain behaviour is observed in other models of juvenile nerve injury: chronic constriction injury (CCI) causes a clear allodynia in adult rats but no change in hindpaw sensitivity when performed at 10 days of age (Howard et al., 2005) and the more proximal injury, spinal nerve ligation, causes only transient allodynia when performed at P14 (Ririe and Eisenach, 2006). This data has been confirmed in other studies (Costigan et al., 2009, Moss et al., 2007, Vega-Avelaira et al., 2009) and recently extended beyond mechanical allodynia to cool and cold allodynia, and altered weight bearing, all of which develop within a few days of adult SNI but are absent following SNI performed at P10 (McKelvey et al., 2015).

This finding is consistent with clinical experience. Neuropathic pain following nerve injury is rare in infants, and only very few reports exist before 5–6 years of age (Anand and Birch, 2002, Howard et al., 2014, Walco et al., 2010) and the incidence of neuropathic pain increases with age at which nerve damage occurs (Atherton et al., 2008). Thirteen years is the median age of onset for paediatric neuropathic pain syndromes, such as phantom pain, complex regional pain syndrome, and peripheral neuropathy pain (Walco et al., 2010) but the reasons for this are not known. Although many of the underlying disease states involving neuropathic pain are less frequent in children, it is also evident that nerve damage is more likely to trigger pain in late childhood and adolescence than at younger ages.

### 1.2 The delayed phase — Late onset neuropathic pain

Infants of all mammalian species tested are capable of displaying robust nociceptive responses to noxious mechanical, thermal and chemical stimulation and develop persistent allodynia and hyperalgesia in response to inflammation of the skin, joints and viscera. So the question remains as to why there is a specific absence of neuropathic pain following nerve injury at younger ages. Clues to this may provide a new insight into this most unpleasant of pain conditions.

Recent longitudinal studies have discovered a novel consequence of juvenile nerve injury. Mechanical hypersensitivity, characteristic of neuropathic pain, does occur but only later in life. While spared nerve injury (SNI) at (P10) has no effect on sensory thresholds in the first 2–3 weeks post-P10 surgery, after that time period, beginning at 21 days post-surgery (P31), the SNI group develop significant hypersensitivity (Vega-Avelaira et al., 2012). This delayed adolescent onset hypersensitivity is also observed using cold stimulation and weight bearing tests but not noxious heat stimulation (McKelvey et al., 2015).

Interestingly, clinical investigation of phantom limb pain in adolescence shows that children with the earliest amputations do develop phantom pain but only after a considerable delay, of a mean of 7 years (Melzack et al., 1997). Furthermore several complex pain syndromes (e.g. CRPS) that emerge in older children are associated with little or no measurable disease activity or inflammation at the time of presentation and are clinically defined as ‘functional’ or ‘medically unexplained’ (Bromberg et al., 2014). This led us to hypothesise that the changes in pain processing over infancy, childhood and adolescence may be of special significance in understanding the maturation of neuropathic pain.

In this review we examine the immediate and longer term changes in dorsal horn nociceptive circuits that follow experimental nerve injury in infant, juvenile and adolescent rats. We present evidence that the clue to the late development of neuropathic pain lies in the maturation of neuroimmune regulation of pain pathways in the spinal cord dorsal horn.

## 2 Cellular effects of nerve injury in early life: The first phase (no pain)

### 2.1 Cell death and compensatory sprouting

In adults, there is some evidence for cell death in the dorsal root ganglia and dorsal horn following nerve injury but this is limited and only observed after a considerable time post-injury (Scholz et al., 2005, Tandrup et al., 2000). The situation is quite different following nerve injury in the immediate postnatal period. Sensory neurons in the rat dorsal root ganglion (DRG) are still undergoing axonal growth and naturally occurring cell death in the first postnatal week and are highly dependent upon their peripheral target skin and muscle for survival (Chong et al., 1992, Coggeshall et al., 1994). As a result, nerve damage in the immediate postnatal period causes substantial and rapid cell death (of up to 75% neurons) in the dorsal root ganglion (Himes and Tessler, 1989, Whiteside et al., 1998, Yip et al., 1984).

The loss of sensory neurons following early nerve damage triggers axonal sprouting within the damaged nerve itself and collateral sprouting of adjacent intact afferent terminals into the denervated region of the dorsal horn. For example, following sciatic nerve section at postnatal day (P) 1, the adjacent saphenous nerve dorsal horn terminal field doubles in size, expanding into areas normally occupied by sciatic nerve terminals, with single afferents growing up to 2000 μm into the deafferented sciatic terminal field (Fitzgerald, 1985, Fitzgerald et al., 1990, Fitzgerald and Vrbová, 1985). Both A fibres and substance P- and CGRP-expressing C fibres are involved in this sprouting (Reynolds and Fitzgerald, 1992, Shortland and Fitzgerald, 1994). The A fibre terminals of surviving axotomized primary afferent neurons also sprout dorsally into lamina II and join the invading intact A and C fibres contributing to the overlap in central representation of nerve territories (Shortland and Fitzgerald, 1994).

Importantly, the new sprouted terminals form functional connections with dorsal horn neurons, such that cells that normally only respond to inputs from the damaged nerves now respond to intact inputs from adjacent body areas. For example, after neonatal sciatic nerve section, dorsal horn neurons with receptive fields in the saphenous skin region can be found throughout L3, L4 and L5 segments, while they are normally restricted to L3 and rostral L4 (Shortland and Fitzgerald, 1991) and stimulation of nearby intact nerves evoke greater fos activation in denervated areas of the dorsal horn (Shortland and Molander, 2000). Nevertheless, loss of primary afferent input apparently withdraws trans-synaptic trophic support from the dorsal horn and projection neurons have substantially fewer primary dendrites and secondary branches compared to controls following neonatal nerve damage (Fitzgerald and Shortland, 1988) accompanied by a small loss of interneurons (Lowrie and Lawson, 2000).

Importantly, these plastic changes only occur if nerve injury is performed in the first days after birth. The need for neurotrophic support from target tissues passes and there is no DRG cell death or compensatory central afferent terminal sprouting, and little somatotopic map reorganisation or transsynaptic trophic changes after the first week of life (Fitzgerald, 1985, Fitzgerald and Shortland, 1988, Shortland and Fitzgerald, 1994). Thus nerve damage in the second postnatal week, at P10 and P21 does not cause any DRG cell loss (Himes and Tessler, 1989, Whiteside et al., 1998) and central afferent sprouting declines sharply with age at the time of nerve damage, being very weak if damage is on day 5, and non-existent if sectioning takes place on day 10 (Fitzgerald, 1985).

These changes cannot therefore explain the lack of neuropathic pain which follows nerve injury performed up to 28 days after birth (Howard et al., 2005, McKelvey et al., 2015).

### 2.2 Glial responses: Weak microglial activation and release of anti-inflammatory cytokines

The absence of neuropathic pain following early life nerve injury has been ascribed to the low level glial response in the dorsal horn of the spinal cord. In adults the role of the immune system in the development and maintenance of neuropathic pain is well documented and is dominated by a rapid onset pro-inflammatory response that leads to the sensitization of neurons in the dorsal horn and pain-like hypersensitivity (Coull et al., 2005, Gao and Ji, 2010, Taves et al., 2013). In contrast to the effects in adults, spared nerve injury at P10 causes little or no increase in the expression of either microglia (MHC-II DMα, MHC-II DMβ, integrin-αM, CD68, IBA-1) or astrocyte (GFAP) markers in the dorsal horn spinal cord (Costigan et al., 2009, Moss et al., 2007, Vega-Avelaira et al., 2007, Vega-Avelaira et al., 2009) and no increase the expression of pro-inflammatory mediators, including TNF, BDNF or IFN-γ in the dorsal horn 7 days after surgery (Costigan et al., 2009, McKelvey et al., 2015). Importantly, intraspinal injections of lipopolysaccharide (LPS) or N-methyl-d-aspartate (NMDA) can activate microglia at this age showing that the innate immune responsiveness of the microglia themselves is not the problem (Moss et al., 2007). This blunted response is also observed in infant rats following other insults such as peripheral nerve C-fibre stimulation, where immune cells mount a weak inflammatory cytokine production associated with fewer protective immune cells compared to adults (Hathway et al., 2009).

The weak microglial response to juvenile nerve injury is accompanied by a distinct dorsal horn cytokine response that differs considerably from that following adult nerve injury. While there is a limited pro-inflammatory response, SNI in infants induces a striking increase in the expression of anti-inflammatory mediators, characterised by an increase in the expression of the transcription factor GATA3, and the cytokines, IL-10 and IL-4 (McKelvey et al., 2015). GATA3 is a regulator of T-cell development and promotes the differentiation of CD4 T-cells into a Th2 cell lineage and the secretion of anti-inflammatory cytokines including IL-4, IL-10, and IL-13 while inhibiting Th1 cell differentiation and IFN-γ production (Ferber et al., 1999, Ouyang et al., 1998). IL-4 signalling induces T-cell proliferation and differentiation into a Th2 phenotype and suppresses macrophage and microglia M1 phenotypes and pro-inflammatory mediator expression (Stein et al., 1992). IL-10 also acts to inhibit pro-inflammatory mediator release as well as reducing the recruitment of immune related glia cells in the spinal cord (Gordon, 2003, Milligan and Watkins, 2009, Ponomarev et al., 2005). The upregulated IL-10, IL-4 and GATA3 are commonly associated with T cells and while resident CD2 + T-cell numbers are not increased following infant nerve injury (Costigan et al., 2009), it is possible that their activity is altered. Furthermore microglia or neurons themselves may be a source of anti-inflammatory cytokines (Milligan and Watkins, 2009, Ponomarev et al., 2005). LPS application to microglia derived from infant spinal cords significantly increases IL-10 release and IL-10 mRNA expression in vitro (Werry et al., 2011).

The dominant anti-inflammatory response following nerve injury does not mean that immature pain circuits are incapable of responding to pro-inflammatory mediators or that they are not released in young spinal cord. Nerve injury sensitizes neonatal lamina I neurons to TNF in vitro (Li and Baccei, 2011) and intrathecal application of TNF and lipopolysaccharide (LPS) activated microglia are both able to overcome the anti-inflammatory activity following nerve injury in infant mice and induce mechanical hypersensitivity (McKelvey et al., 2015). Thus infant rodents are able to respond to stressors such as TNF or LPS-activated microglia (which can release TNF to sensitize dorsal horn neurons and enhance pain states (Berta et al., 2014)) and infant spinal microglia are clearly activated by peripheral inflammatory stimulation (Vega-Avelaira et al., 2013) but following nerve injury, microglia are apparently stopped from producing TNF and BDNF by a nerve injury-induced anti-inflammatory response.

The infant anti-inflammatory response does not require actual nerve damage; it can also be evoked by brief stimulation of intact afferent C fibre nociceptors in infants (McKelvey et al., 2015). The same C fibre stimulation in adults induces pain hypersensitivity and microglia activation in the spinal cord, that are attenuated by pre-treatment with minocycline (Hathway et al., 2009), an inhibitor of pro-inflammatory polarised microglia (Kobayashi et al., 2013), thus transiently mimicking changes in the dorsal horn that underpin chronic pain states (Taves et al., 2013). The fact that C-fibre stimulation in infants stimulates expression of anti-inflammatory IL-10 and IL-4 (McKelvey et al., 2015), suggests that their release is directly activated by neurotransmitter release from C fibre terminals in the dorsal horn and that the response following infant nerve injury could be a direct result of activity in damaged C fibre afferent terminals.

## 3 Cellular effects of nerve injury in early life: The delayed phase (late onset pain)

### 3.1 Glial responses: Increased microglial activation and release of pro-inflammatory cytokines

Longitudinal studies have shown that following nerve injury at postnatal day (P) 10 there is a delayed onset of pain hypersensitivity (McKelvey et al., 2015, Vega-Avelaira et al., 2012) and that this is accompanied by increased microglial activation and NMDA dependent central sensitization of spinal nociceptive circuits, but no significant change in dorsal horn p38 or JNK expression. Pre-emptive minocycline administered daily (from before P10 surgery until P21), does not prevent the effect, perhaps because minocycline is a p38 inhibitor, and p38 does not to play a part in this process (Garrido-Mesa et al., 2013, Vega-Avelaira et al., 2012). On the other hand ketamine produces a dose-dependent reversal of hypersensitivity to mechanical stimulation (Vega-Avelaira et al., 2012), suggesting that the onset of hypersensitivity is due to a maintained NMDA mediated central sensitization. The delayed mechanical hypersensitivity coincides with an increase in the expression of immune cell markers (microglia and T-cells), and expression pro-inflammatory mediators including TNF and BDNF that can alter synaptic transmission in the dorsal horn and are associated with pain sensitization (Coull et al., 2005, Taves et al., 2013). In addition, the anti-inflammatory mediators (IL-10 and IL-4) that were upregulated in the early post-nerve injury phase and can act in a regulatory capacity to control inflammation, are now reduced to sham control levels (McKelvey et al., 2015).

Thus, as the nerve injured infants grow up, the anti-inflammatory response changes to a proinflammatory response; levels of the anti-inflammatory mediators IL-4 and IL-10 return to control levels and GATA3 falls transiently below control levels, whereas expression of proinflammatory markers, IBA1, BDNF, and TNFα increase. Notably, the switch to a proinflammatory response coincides with a delayed onset of mechanical hypersensitivity and a significant increase in spontaneous, tactile and acetone-evoked activity in dorsal horn neurons, similar to that seen following adult nerve injury (McKelvey et al., 2015). Because the levels of GFAP do not increase at this time, astrocyte activation is unlikely to drive delayed-onset hypersensitivity. The timing is entirely consistent with the reported postnatal change in toll-like receptor (TLR) inducible cytokine and chemokine release from microglia, which is at its lowest at postnatal day 21 but rises considerably towards adolescence (Scheffel et al., 2012). Thus, despite the fact that the nerve injury was performed in infancy, pain behaviour emerges at adolescence, at least in part as a result of a change in neuroimmune activity.

### 3.2 Delayed neuropathic pain can be ‘unmasked’ at an earlier age by suppressing anti-inflammatory cytokine function

We have recently shown that the upregulated anti-inflammatory cytokines during the early phase following infant nerve injury actively suppresses neuropathic pain in the weeks following surgery. Blockade of IL-10 activity with intrathecal functional blocking antibody for three consecutive days (7–9 days post-surgery at P10) causes a significant hypersensitivity to mechanical stimulation in nerve injured but not sham operated mice. Thresholds recovered 24 h after the last injection. Intrathecal injection of the control antibody (IgG) into infant SNI mice did not induce significant changes in mechanical thresholds. In adults, intrathecal administration of anti-IL-10 after SNI has no significant effect on mechanical thresholds at any time (McKelvey et al., 2015).

Thus the release of IL-10 following nerve injury is functionally essential for the absence of mechanical hypersensitivity. Neuropathic pain is therefore not really absent in the early phase, but actively suppressed and can be unmasked if the anti-inflammatory response is blocked. Exogenous IL-10 and IL-4 are anti-nociceptive in adult neuropathic models, suppressing proinflammatory cytokines, microglia responses and pain behaviour (Milligan et al., 2005, Milligan and Watkins, 2009) and our results show that in infants this antinociception naturally dominates following nerve injury (McKelvey et al., 2015).

## 4 What triggers delayed onset neuropathic pain?

### 4.1 A natural transient developmental role for anti-inflammatory activity in the CNS

**Fig. 1:**
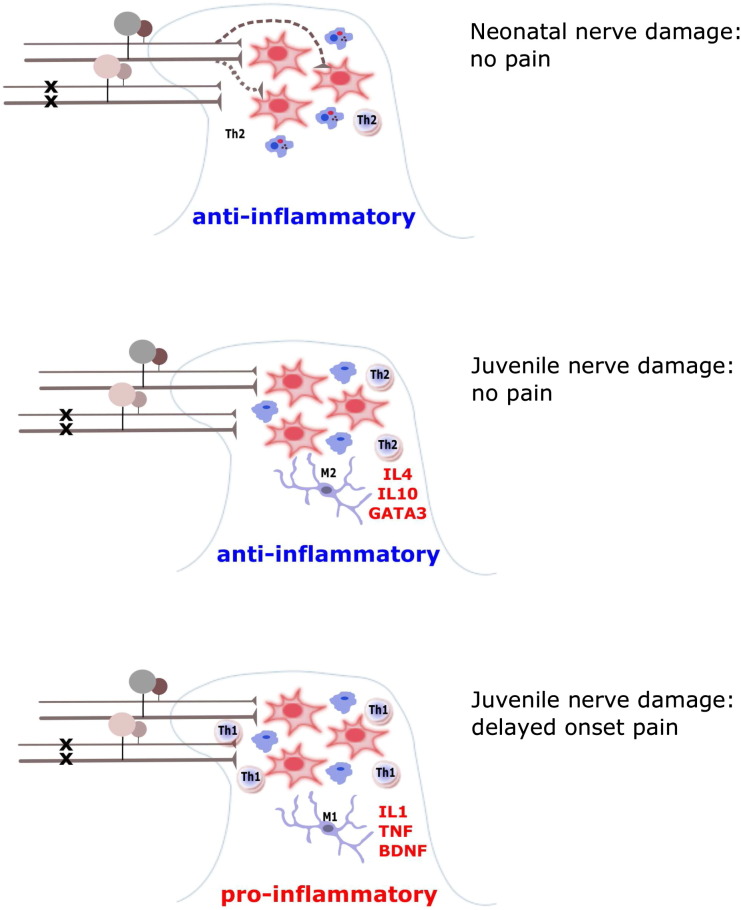
The dorsal horn response to peripheral nerve injury depends upon age. A model is proposed above of the cellular activity in the dorsal horn following nerve injury at different time points and postnatal stages Top: Neonatal (day 1–7) nerve injury results in primary afferent sprouting, and there is no pain behaviour. It is proposed that microglia are phagocytic (blue cells) and resident T helper cells (pink circular cells) are predominantly type Th2, that is anti-inflammatory. This cellular environment promotes and supports structural plasticity and postnatal neuronal circuit development (dark red neurons). Centre: Juvenile (day 10) nerve injury initially triggers an anti-inflammatory response in the dorsal horn with anti-inflammatory T helper cell activity (Th2) and microglial activity (M2) associated with release of cytokines such as IL4 and IL10. There is no pain behaviour. Bottom: Juvenile (day 10) nerve injury causes a later, delayed onset pro-inflammatory response in the dorsal horn. This arises from a switch of microglial and T cell activity from M2 and Th2 to M1 and Th1, associated with the release of pro-inflammatory cytokines such as IL1, TNF and the neurotrophin, BDNF. These, in turn, excite dorsal horn neurons and as a result trigger pain behaviour.

It appears then, that neuropathic pain can emerge later in life, following earlier nerve injury due to a natural developmental change in the neuroimmune system (Fig. 1). In the first weeks of life, glial cell activity within the dorsal horn is tailored to the natural requirements of a growing neural circuit. Microglia are the principal resident innate immune cells in the central nervous system (CNS) and are essential during the early development for synaptic pruning, developmental neuronal apoptosis and remodelling (Paolicelli et al., 2011, Schafer et al., 2012). Microglia undergo striking transformations in both morphology and activity during development (Harry, 2013, Harry and Kraft, 2012) displaying an activated morphology and high phagocytic activity during the postnatal period in contrast to the low mitotic activity and highly motile processes of adult microglia, that constantly survey their microenvironment. Microglia also express higher levels of iNOS, TNFα and arginase-I mRNA in early postnatal development compared to the adult CNS (Crain et al., 2013), suggesting that their activities in the developing CNS may be distinct from those in the adult. Microglial cell numbers increase within the first 2 postnatal weeks and decline in the third week; microglial density in the postnatal CNS is two times higher than in the adult. This supports the notion that during the early postnatal period, the primary function of microglia may be to participate in “building” the CNS, whereas in adulthood, microglia switch to a maintenance/surveillance mode (Nikodemova et al., 2015).

The recently discovered novel anti-inflammatory response to nerve injury in young rats and mice (McKelvey et al., 2015) is consistent with the ‘default’ immune response in neonates which is skewed in an anti-inflammatory direction (Adkins, 2000, Elahi et al., 2013, PrabhuDas et al., 2011). It is increasingly recognised that the immune profile in both peripheral (T-cells) and central residing immune cells (microglia) undergo a developmental shift with age from an anti-inflammatory skewed response (such as high IL-10) to pro-inflammatory in both rodents and humans (Elahi et al., 2013, PrabhuDas et al., 2011, Shigemoto-Mogami et al., 2014). For example cytokine analysis of LPS-activated microglia between P0 to P49 mice reveal a gradual developmental shift to a defence-orientated M1 phenotype, with an increasing induction of TNF-α, IL-1β, IL-6 and a decline in arginase with age (Scheffel et al., 2012). In addition, following both LPS and non-TLR mediated challenges discrete subsets of microglia secrete TNFα with increasing age indicating a shift towards microglia response heterogeneity (Scheffel et al., 2012).

### 4.2 A shift in neuroimmune regulation as nociceptive circuits mature

The function of a predominant anti-inflammatory response observed during this postnatal period in response to nerve injury and other insults may have evolved to prevent excessive inflammation following the transition from a sterile *in utero* setting to colonization with commensal microbes (Maynard et al., 2012). In addition, studies in the healthy brain indicate a dual role for microglia during postnatal development associated with the substantial changes that occur in the connectivity of the CNS over this period. This includes their ability to remove cellular debris and dead cells via phagocytosis activity and secondly, to establish contacts with synapses and regulate the size of dendritic spines during critical periods (Arnoux et al., 2014, Schafer et al., 2012). For example, microglia within the juvenile visual cortex can modify their association with dendritic spines in response to changes in visual sensory experience and actively engulf synaptic structures and exert a major role in controlling the number of synapses through synaptic pruning (Tremblay et al., 2010). This is confirmed by the finding that disruption in microglial function, such as depletion of microglia CX3CL1 receptor or administration of a microglial inhibitor, results in delayed synaptic pruning and maturation of hippocampal synaptic circuits (Paolicelli et al., 2011, Schafer et al., 2012). As substantial rearrangement and refinement of synaptic connections occur in the first weeks in the dorsal horn (Fitzgerald, 2005), it is possible that dorsal horn microglia are enabling the refinement and rearrangement of synaptic connections and pruning of axonal projections, temporarily increasing the production of damage associated molecular patterns (DAMP). In other words, the anti-inflammatory response to nerve damage in infants may be the indirect consequence of the requirements for normal postnatal development in the dorsal horn (Beggs et al., 2002, Bremner and Fitzgerald, 2008, Koch et al., 2012). As a result, a peripheral nerve injury at P10, activates signalling pathways associated with a default anti-inflammatory immune response in the dorsal horn spinal cord and the absence of pain like behaviour, enabling normal postnatal development in the dorsal horn to occur through phagocytic activity, without the initiation of an extensive and damaging inflammatory response and associated hypersensitivity.

### 4.3 An explanation for ‘unexplained’ pain in adolescence?

In this review we have argued that, depending on the developmental status of the animal, nerve injury will trigger different immune responses in the dorsal horn spinal cord (Fig. 1). In very young animals, equivalent to preterm infants, the response to nerve injury is cell death in the DRG and compensatory sprouting and reorganisation of the somatosensory and pain system. Microglial are permissive and their main duty is phagocytosis at this time. Later, in animals aged P10–P21, equivalent to infancy and childhood, this neural plasticity is lost but the nociceptive circuits are still undergoing important maturation facilitated by permissive immune system. At this age, nerve injury evokes an anti-inflammatory immune response, characterised by the release of the anti-inflammatory mediators, IL10 and IL4, which are also released at this age by peripheral nerve C-fibre stimulation. The anti-inflammatory cascade in the dorsal horn suppresses neuronal sensitization, and so pain is naturally suppressed in the early period following infant nerve injury. However, as the animal grows up towards adolescence, a gradual developmental change occurs in the balance of neuroimmune regulation towards a pro-inflammatory response, perhaps to protect against foreign invasion, which dominates and allows the expression of neuronal sensitization and the appearance of pain like behaviour. Important other contributors, yet to be tested, could be stress, that can increase the likelihood of maladaptive immune responses (Graham et al., 2006), and sex differences in glial function contributing to distinct windows of adolescent vulnerability in females (Schwarz and Bilbo, 2012). If the data translates to man, it suggests that nerve injury in early life may go unnoticed at the time of injury, but could emerge in later life as an ‘unexplained’ or ‘functional’ pain. Thus adolescent onset complex pain behaviours may be attributable to unreported injury in early life.
